# Long-term TNF-alpha therapy for preserving beta cell function in new onset type 1 diabetes: a case report

**DOI:** 10.1186/s40842-024-00185-6

**Published:** 2024-09-10

**Authors:** Adya Rao, Lauren M Quinn, Parth Narendran

**Affiliations:** 1 Department of Diabetes, Queen Elizabeth Hospital, University Hospitals of Birmingham, Birmingham, UK; 2 Institute of Immunology and Immunotherapy, University of Birmingham, Birmingham, UK

**Keywords:** Immunotherapy, Immunoprevention, Type 1 diabetes, T1D; Infliximab, TNF-alpha, Tumour necrosis factor alpha, Anti-TNF-α

## Abstract

**Background:**

Type 1 diabetes mellitus (T1D) is an autoimmune disease caused by destruction of pancreatic islet beta-cells. There is significant residual beta-cell function, measured through circulating C-peptide, present at the time of T1D diagnosis but this subsequently decreases with time. Higher residual beta-cell function at diagnosis associates with better glycaemic control and less glucose variability, and later in the disease course with less hypoglycaemia, lower glucose variability and fewer microvascular complications. There is therefore value in preserving residual beta cell function in new onset T1D Immunotherapeutic agents can protect residual beta-cell function in type 1 diabetes. However, clinical trials of such agents, whilst demonstrating C-peptide preservation in short term studies, have yet to be taken forward into routine clinical care due to concerns around safety and long-term efficacy. Here we report the case of a gentleman with newly diagnosed T1D whose glycaemic control and insulin requirement improved whilst on a five year infusion programme of infliximab, a monoclonal antibody against TNF-alpha, for colitis.

**Case presentation:**

A 52-year-old White Caucasian man was diagnosed with T1D in August 2018. Glucose was 25.6 mmol/L, HbA1c was 98mmol/mol and GAD antibodies were strongly positive. HbA1c marginally improved to 91mmol/mol following initiation of insulin detemir 5 units at night and 1:10 g of insulin aspart (November 2018). In June 2019, he developed rectal bleeding and abdominal pain. Following colonoscopy, he was diagnosed with “indeterminate colitis” and commenced on 6-weekly infusions of 400-450 mg infliximab. Thus far, he has received 32 doses and achieved colitis remission. Following infliximab initiation there was increased frequency of mild-moderate hypoglycaemia and he was gradually weaned off and discontinued detemir in June 2020. Since then, HbA1c improved from 57mmol/mol in August 2019 to 52mmol/mol in April 2022, remaining stable at 51mmol/mol. His most recent HbA1c is 54mmol/mol in February 2024. His c-peptide was 550pmol/L in October 2022 and 442pmol/L in February 2024, suggesting well-preserved beta-cell function almost 6 years post-diagnosis.

**Conclusions:**

Our patient’s improvement in glycaemic control can be explained by immunomodulation and C peptide preservation from infliximab. With the growing focus on type 1 diabetes disease modulation and working towards an ‘insulin free T1D’, our findings strengthen the evidence base for the repurposing of and long-term treatment with anti-TNF-α agents to preserve beta-cell function in new onset T1D.

**Supplementary Information:**

The online version contains supplementary material available at 10.1186/s40842-024-00185-6.

## Background

Type 1 diabetes (T1D) results from the autoimmune destruction of pancreatic-islet insulin-secreting beta cells [1]. Whilst characterised by significant beta cell loss, some residual beta cell function remains at diagnosis and this subsequently falls off with continued autoimmune attrition [2–4]. Beta cell function is measured as circulating C peptide, a fragment of the insulin precursor molecule. C peptide levels in adults newly diagnosed with T1D can be over 1000pmol/L, falling to less than 200pmol/L within three years of diagnosis [5]. Residual beta cell function at diagnosis associates with appreciable clinical benefits. The benefits include lower glucose levels and less glucose variability near the time of diagnosis. Later in the course of disease, residual beta cell function associates with less hypoglycaemia, lower glucose variability and fewer microvascular complications such as retinopathy, neuropathy, and nephropathy [6]. There is therefore a strong clinical argument for preserving residual beta cell function in people newly diagnosed with T1D [5].

Immune modulating therapies can preserve beta cell function if administered sufficiently early after T1D diagnosis [7]. A mature immune response against the pancreatic islet involves different cell types and immune pathways and modulating this potentially attenuates beta cell destruction [3]. Tumour necrosis factor alpha (TNF-α) is a cell-cell signalling molecule, a cytokine, involved in immune cell survival and proliferation. Inhibiting TNFa action prevents T1D in animal models [8]. Two studies in patients with newly diagnosed T1D have also been promising. A 2009 pilot randomised controlled trial (RCT) of etanercept in children with new onset T1D demonstrated improved residual beta cell function (measured as C-peptide) and glucose control (measured as glycated haemoglobin HbA1c) [9]. Etanercept acts as a soluble TNF receptor, binding TNF-α and inhibiting its action [10]. More recently golimumab administration in children and young adults with newly diagnosed T1D resulted in increased endogenous insulin production and decreased exogenous insulin use [11]. Golimumab is a humanised monoclonal antibody to TNFa thus blocking the biological activity this cytokine.

Whilst promising, these studies of anti-TNF therapies are of short duration and therefore provide limited information on long-term outcomes. Here we describe a patient who developed inflammatory bowel disease (IBD) requiring treatment with infliximab, a chimeric monoclonal antibody against TNF-α [12] around the time of developing T1D. He continued on infliximab long-term, thus providing insight into the long-term acceptability and efficacy of anti-TNF therapy in T1D.

### Case presentation

A 52-year-old Caucasian man presented in August 2018 to primary care with polydipsia, polyuria and approximately 3 kg of weight loss. He had a history of autoimmune disease, including uveitis and ankylosing spondylitis, and atopy (eczema and asthma). At presentation, his glucose was 25.6mmol/L, HbA1c was 98mmol/mol and he had strongly positive GAD antibodies of 1321.6IU/ml. He was initially treated with metformin 500 mg BD and gliclazide 40 mg OD, but due to persistently elevated blood sugars (11–15 mmol/L) he was referred to the diabetes specialist clinic in October 2018, where his HbA1c was found to have increased to 118mmol/mol. Here, a diagnosis of T1D was made and he was commenced on detemir 5 units at night and insulin aspart titrated to 1 unit for every 10 g of carbohydrates. His HbA1c marginally improved to 91 mmol/mol in November 2018.

In May 2019, nine months after presentation with T1D, he developed intermittent bloody diarrhoea and abdominal pain. A colonoscopy showed patchy, moderate acute and chronic inflammation in the rectum, and patchy acute inflammation and focal ulceration in the colon, with no evidence of active inflammation, dysplasia or neoplasia in the ileum. His faecal calprotectin was 1939ug/g and he had a negative Quantiferon TB assay. A diagnosis of indeterminate colitis was made and he was treated initially with IV hydrocortisone and then a reducing dose of oral prednisolone. He required 3 doses of rescue therapy with infliximab and was commenced on mesalazine 2.4 g BD on discharge. Shortly after his first acute colitis flare, he was commenced on azathioprine 75 mg OD and 6-weekly infliximab infusions with his first outpatient infusion administered in July 2019. He responded very well to his colitis treatment and achieved clinical remission in October 2019 (CRP 2 and faecal calprotectin 103ug/g).

Over the course of the next year, during which he was on regular infliximab infusions, he experienced increased frequency of mild-moderate hypoglycaemia. As such, his insulin requirement decreased, with detemir gradually reduced until it was no longer required, while insulin aspart was continued at 1:10 g until present.

Azathioprine was eventually stopped in August 2022 because the patient developed a superficial BCC [13] and mesalazine was stopped in January 2023 due to an itchy rash on the torso, face and buttock. The frequency of infliximab infusions was decreased from 6-weekly to 8-weekly in September 2021. To date he has received 33 infliximab infusions of 400-450 mg (excluding those for his first colitis flare as an inpatient). He continues to receive infliximab infusions 8-weekly, with his last infusion being on 11/01/2024. He stated that these infusions are “easy to have” as he “is only in there for an hour” and he has “had no side effects from them” thus far.

**Fig. 1:**
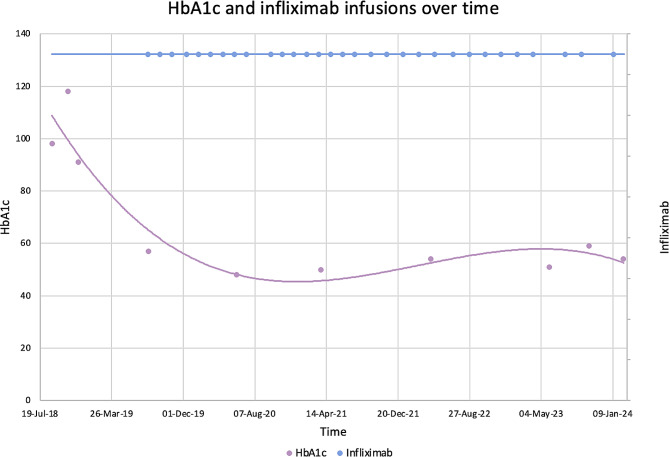
Timeline of glucose control and infliximab infusions. HbA1c at diagnosis in August 2018 was 98mmol/mol, after which he was treated with metformin and gliclazide. Despite treatment, HbA1c rose to 118mmol/mol in October 2018, after which he was commenced on detemir and insulin aspart. HbA1c dropped slightly to 91mmol/mol in November 2018 on this treatment. The next available HbA1c is 57mmol/mol in August 2019, having received 3 infliximab infusions for his acute colitis flare in July 2019. His most recent HbA1c is 54mmol/mol in February 2024

Figure 1 shows the timeline of his infliximab infusions and his glucose control. His C peptide was 550pmol/L in October 2022 and 442pmol/L in February 2024.

## Discussion and conclusions

We describe a case of adjunctive treatment of new-onset T1D with anti-TNF therapy which resulted in lower insulin requirements, better glucose control and preserved C peptide over a 5-year period. Our case supports the findings of the two short-term RCTs previously alluded to [9, 11] as well as that of another case report where treatment with infliximab resulted in resurgence of insulin secretion and lower insulin requirements over a one-year period [14]. These studies are summarised in an additional file (Supplementary Table 1, Additional File 1). Our case however goes further to demonstrate that these benefits can persist long-term, and that the treatment can be acceptable without undue side-effects.

The main strength of our study is the long-term follow up of the case and available documentation of the timeline of insulin and anti-TNF therapies. It is weakened by the lack of sequential C peptides, including a C peptide measure at diagnosis with T1DM.

Our patient’s C-peptide was 442pmol/L in February 2024, 5.5 years post-T1D diagnosis which is unusually high for his stage of disease [15]. This suggests that infliximab’s benefit was mediated through beta cell preservation. Our findings demonstrate the protective effect of repeated infliximab infusions over an extended period (over 5 years) on glycaemic control and insulin requirement in newly diagnosed T1D. On an individual level, these benefits translate to a reduction in the practical burdens associated with insulin such as continuous monitoring, multiple injections and risk of hypoglycaemia. A reduction in insulin requirements also facilitates maintenance of healthy weight and associated reduction in cardiovascular risk. Furthermore, higher C peptide levels early in T1D associates with lower rates of long-term vascular complications [16].

The fall in insulin requirements after initiation of infliximab therapy is interesting and may be mediated through either recovery of beta cell function or increased insulin sensitivity. There is some evidence that TNF mediates resistance to the actions of insulin [17], and therefore anti-TNF therapy would potentially increase insulin sensitivity and reduce insulin requirements [14]. Insulin sensitivity was not formally measured in our case, and it is therefore difficult to estimate its contribution to the reduced insulin requirements.

On a broader scale, our findings support the case for immunotherapy for beta cell preservation in newly diagnosed T1D. Several RCTs have demonstrated the efficacy of different immunotherapeutic agents for preserving beta cell function in new onset T1D [18], but these have yet to be taken forward to clinical practice. These studies have all been less than two-year duration and long-term follow up data is difficult to obtain in a RCT setting. Therefore, case reports such as these help understand the role of these therapies in clinical care.

Immunotherapy to preserve beta cells, if administered before the requirement for insulin and when there is appreciable beta cell function, can delay symptomatic T1D. Such therapies are now becoming licensed – tepelizumab, an anti-CD3 antibody, was licensed in the United States in November 2022 for individuals aged over 8 years with stage 2 type 1 diabetes [3, 19], and it is currently undergoing NICE consultation in the UK [20]. Our case report suggests than in addition to immunoprevention, immunotherapy at diagnosis with symptomatic T1D (Stage 3) [3] may also be worth pursuing.

## Electronic supplementary material

Below is the link to the electronic supplementary material.

Supplementary Material 1

## Data Availability

The dataset(s) supporting the conclusions of this article is(are) included within the article.
