# Correction: Long-term TNF-alpha therapy for preserving beta cell function in new onset type 1 diabetes: a case report

**DOI:** 10.1186/s40842-025-00217-9

**Published:** 2025-02-21

**Authors:** Adya Rao, Lauren M. Quinn, Parth Narendran

**Affiliations:** 1 Department of Diabetes, Queen Elizabeth Hospital, University Hospitals of Birmingham, Birmingham, UK; 2 Institute of Immunology and Immunotherapy, University of Birmingham, Birmingham, UK

**Correction: Clin Diabetes Endocrinol 10, 26 (2024)**

10.1186/s40842-024-00185-6

Following publication of the original article [1], the statements under Ethics approval and consent to participate and Consent for publication sections are need to swap around.

Currently it reads.

**Ethics approval and consent to participate**

Informed consent was obtained from the patient whose case we report, in accordance with the declaration of Helsinki.

**Consent for publication**

Not applicable.

And we would need the two statements to be swapped around:

**Ethics approval and consent to participate**

Not applicable.

**Consent for publication**

Informed consent was obtained from the patient whose case we report, in accordance with the declaration of Helsinki.

The original article has been corrected.
