# Supplementary material for: Long-term TNF-alpha therapy for preserving beta cell function in new onset type 1 diabetes: a case report
Source: Clin Diabetes Endocrinol. 2024 Sep 10;10:26. doi: 10.1186/s40842-024-00185-6 (PMC11386087; doi:10.1186/s40842-024-00185-6)
Supplement: Supplementary file 1 — Supplementary Material 1 [file 40842_2024_185_MOESM1_ESM.docx]

**Supplementary table 1: Summary of existing literature on anti-TNF therapy for newly diagnosed T1DM**

| **Study type** | **Year of publication** | **Population** | **Intervention** | **Control** | **Follow-up period** | **Results** | **Conclusions** |
| --- | --- | --- | --- | --- | --- | --- | --- |
| Case report^1^ | 2000 | 7-year-old girl with 3-year history of juvenile rheumatoid arthritis (RA) and positive family history of T1DM (maternal aunt) | Etanercept infusions 0.4mg/kg twice-weekly commenced in March 1999 (3 years post- RA diagnosis) | N/A | 4 years | 1. Patient developed T1DM 5 months after commencing etanercept- presented with DKA and tested positive for GAD antibodies (3.7 units/ml). Etanercept discontinued and subcutaneous insulin commenced on discharge. 2. GAD antibodies were positive pre-etanercept and during etanercept treatment. | Proposed that anti-TNF therapy, in particular etanercept, may expedite development of T1DM in predisposed individuals (GAD antibody positive) with juvenile rheumatoid arthritis |
| Case report^2^ | 2006 | 55-year-old female with 25-year history of T1DM and 22-year history of rheumatoid arthritis (RA) | Etanercept 25mg twice-weekly commenced in 2003 for refractory RA; (19 years post RA diagnosis)  Adalimumab commenced in 2004 (20 years post RA diagnosis) - dose unspecified | N/A | N/A | 1. Erratic BMs and severe hypoglycaemic attack without warning within 3 months of commencing etanercept, following previously stable diabetes. Glycaemic control stabilised once etanercept stopped. 2. Hypoglycaemic attack 12 hours following adalimumab administration. Adalimumab stopped. | Anti-TNF agents (etanercept and adalimumab) resulted in hypoglycaemic attack in patient with longstanding T1DM and RA; proposed that this is potentially mediated by increased insulin sensitivity |
| Case report^3^ | 2009 | 35-year-old woman with 20-year history of rheumatoid arthritis and FH of T1DM (mother) | Etanercept infusions 25mg twice-weekly commenced in 2000 (11 years post-RA diagnosis) | N/A | 9 years | 1. Patient developed T1DM 3 years after commencing etanercept infusions. Presented with polydipsia, polyuria, fatigue and weight loss. Glucose >25mmol/L, HbA1c 11.6%, GAD 1312 IU/ml (raised) 2. GAD antibodies were detected in 1997 pre-etanercept treatment; titres increased 10-fold during the course of etanercept infusions | 1. Anti-TNF-α therapy did not prevent the development or the progression of T1DM in patient with longstanding RA 2. Anti-TNF therapies proposed to be ineffective for prevention of T1DM |
| Double-blind randomised controlled trial^4^ | 2009 | N=18  Inclusion criteria- male and female subjects aged 3–18 years with type 1 diabetes, GAD-65 and/or islet cell antibody positivity, A1C >6% at diagnosis, insulin regimen with three injections daily, white blood cell count between 3,000 and 10,000 and platelets >100,000, normal alanine aminotransferase and aspartate aminotransferase, creatinine <1.8 mg/dl, and type 1 diabetes duration ≤4 weeks | Etanercept – twice-weekly subcutaneous injections dosed at 0.4 mg/kg up to max. dose 25 mg/dose | Three-injection insulin regimen:  Humalog and NPH before breakfast, Humalog before dinner, and NPH at bed time. | 24 weeks followed by 12-week washout period | 1. Lower HbA1c at week 24 in the etanercept group (5.91 ± 0.5%) compared with placebo group (6.98 ± 1.2%; P < 0.05) 2. Higher percentage decrease from baseline in HbA1c in etanercept group vs. placebo (etanercept 0.41 ± 0.1 vs. placebo 0.18 ± 0.21; P < 0.01) 3. Higher percentage change in C-peptide AUC from baseline in etanercept vs. placebo (39% increase in etanercept group, 20% decrease in placebo group, p<0.05) 4. Greater decrease in insulin dose from baseline to week 24 in etanercept vs. placebo (18% decrease in etanercept group vs 23% increase in placebo group (p<0.05) | Etanercept treatment in paediatric patients with newly diagnosed diabetes resulted in lower HbA1c and increased endogenous insulin production, suggesting beta-cell preservation |
| Case report^5^ | 2013 | 29-year-old Caucasian man with newly-diagnosed T1DM | IV infliximab (5 mg/kg body wt) at weeks 0, 2, and 6 then every 8 weeks thereafter  Regime commenced 9 months after T1DM diagnosis | N/A | 1 year | 1. Immediate and sustained 2.4-fold increase in insulin secretion over period of infliximab administration (measured via “c-peptide secretion index” defined as [(plasma C-peptide at 30 min of mixed-meal test) – (fasting plasma C-peptide)]/fasting plasma glucose 2. Progressive up to 6.9-fold increase in insulin sensitivity over period of infliximab administration (measured via “whole-body insulin sensitivity index” defined as 10,000/√(fasting plasma glucose × fasting plasma C-peptide × mean mixed-meal test plasma glucose × mean mixed-meal test plasma C-peptide) 3. No change in HbA1c with infliximab infusions 4. Decrease in insulin lispro requirements from 0.8 to 0.5 units per 10g carbohydrates after starting infliximab | Improved insulin production and insulin sensitivity, and reduction in basal insulin requirements, with infliximab infusions in young man with newly diagnosed T1DM |
| Double blind placebo-controlled, parallel-group trial^6^ | 2020 | N=84 (56 golimumab group, 28 placebo group)  Inclusion criteria- aged between 6 and 21 years, diagnosis of type 1 diabetes according to American Diabetes Association (ADA) criteria, able to undergo randomization within 100 days after diagnosis, peak C-peptide level of at least 0.2 pmol per milliliter after a 4-hour mixed-meal tolerance test, at least one autoantidboy positive (anti–glutamic acid decarboxylase 65, anti–islet antigen 2, anti–zinc transporter 8, anti–islet-cell antigen, or anti-insulin) | Golimumab subcutaneous injections-  Induction dose at weeks 0 and 2 - 60mg/m^2^ if body weight <45kg and 100mg if body weight >45kg.  Maintenance dose every 2 weeks – 30mg/m^2^ if body weight <45kg and 50mg if body weight >45kg | N/A | 52 weeks | 1. Higher mean (+/-SD) 4-hour C-peptide AUC at week 52 in golimumab group vs placebo (0.64±0.42 pmol per milliliter vs. 0.43±0.39 pmol per millliter, P<0.001). 2. Less percentage decrease in 4-hour C-peptide AUC from baseline in golimumab group vs placebo (12% vs 56%, p<0.001) 3. No significant difference in HbA1c at week 52 between golimumab and placebo 4. Less exogenous insulin use in golimumab group vs placebo at week 52 (0.51 units/kg/day in golimumab group vs 0.69 units/kg/day in placebo group, p=0.001) 5. No significant difference in mean number of hypoglycaemic events between golimumab and placebo | Golimumab resulted in better endogenous insulin production and less exogenous insulin use in children and young adults with newly diagnosed T1DM |

**References**

1. Bloom BJ. Development of diabetes mellitus during etanercept therapy in a child with systemic-onset juvenile rheumatoid arthritis. Arthritis & Rheumatism. 2000;43(11):2606-8.

2. Boulton JG, Bourne JT. Unstable diabetes in a patient receiving anti-TNF-alpha for rheumatoid arthritis. Rheumatology (Oxford). 2007;46(1):178-9.

3. Tack CJ, Kleijwegt FS, Van Riel PL, Roep BO. Development of type 1 diabetes in a patient treated with anti-TNF-alpha therapy for active rheumatoid arthritis. Diabetologia. 2009;52(7):1442-4.

4. Mastrandrea L, Yu J, Behrens T, Buchlis J, Albini C, Fourtner S, et al. Etanercept Treatment in Children With New-Onset Type 1 Diabetes: Pilot randomized, placebo-controlled, double-blind study. Diabetes Care. 2009;32(7):1244-9.

5. Timper K, Hruz P, Beglinger C, Donath MY. Infliximab in the treatment of Crohn disease and type 1 diabetes. Diabetes Care. 2013;36(7):e90-1.

6. Quattrin T, Haller MJ, Steck AK, Felner EI, Li Y, Xia Y, et al. Golimumab and Beta-Cell Function in Youth with New-Onset Type 1 Diabetes. New England Journal of Medicine. 2020;383(21):2007-17.
